# Using Rainfall and Temperature Data in the Evaluation of National Malaria Control Programs in Africa

**DOI:** 10.4269/ajtmh.16-0696

**Published:** 2017-09-27

**Authors:** Madeleine C. Thomson, Israel Ukawuba, Christine L. Hershey, Adam Bennett, Pietro Ceccato, Bradfield Lyon, Tufa Dinku

**Affiliations:** 1 International Research Institute for Climate and Society, Palisades, New York;; 2 Department of Environmental Health Sciences, Mailman School of Public Health, Columbia University, New York, New York;; 3 President's Malaria Initiative, United States Agency for International Development, Washington, District of Columbia;; 4 Malaria Elimination Initiative, Global Health Group, University of California, San Francisco, California

## Abstract

Since 2010, the Roll Back Malaria (RBM) Partnership, including National Malaria Control Programs, donor agencies (e.g., President's Malaria Initiative and Global Fund), and other stakeholders have been evaluating the impact of scaling up malaria control interventions on all-cause under-five mortality in several countries in sub-Saharan Africa. The evaluation framework assesses whether the deployed interventions have had an impact on malaria morbidity and mortality and requires consideration of potential nonintervention influencers of transmission, such as drought/floods or higher temperatures. Herein, we assess the likely effect of climate on the assessment of the impact malaria interventions in 10 priority countries/regions in eastern, western, and southern Africa for the President's Malaria Initiative. We used newly available quality controlled Enhanced National Climate Services rainfall and temperature products as well as global climate products to investigate likely impacts of climate on malaria evaluations and test the assumption that changing the baseline period can significantly impact on the influence of climate in the assessment of interventions. Based on current baseline periods used in national malaria impact assessments, we identify three countries/regions where current evaluations may overestimate the impact of interventions (Tanzania, Zanzibar, Uganda) and three countries where current malaria evaluations may underestimate the impact of interventions (Mali, Senegal and Ethiopia). In four countries (Rwanda, Malawi, Mozambique, and Angola) there was no strong difference in climate suitability for malaria in the pre- and post-intervention period. In part, this may be due to data quality and analysis issues.

## INTRODUCTION

Since 2010, members of the Roll Back Malaria (RBM) Partnership, including National Malaria Control Programs (NMCPs), donor agencies such as the President's Malaria Initiative (PMI), and Global Fund (GF), and other stakeholders have been evaluating the impact of scaling up malaria control interventions on all-cause under-five mortality, malaria prevalence, and malaria cases in specific countries in sub-Saharan Africa (SSA). The theoretical evaluation framework proposed by RBM Monitoring and Evaluation Reference Group^1^ recommends correctly measuring and accounting for non-malaria program factors, such as rainfall, to tease out the potential association(s) in the causal pathway between these factors, and all-cause under-five mortality. This has been reiterated in the practitioners framework outlined by Ye and others in this special issue.^2^

In SSA, where the transmission of malaria is mainly supported by vectors of the *Anopheles gambiae* species complex,^3^ the spatial and temporal dynamics of malaria transmission (in the absence of control) is substantially determined by the spatial and seasonal patterns of the prevailing rainfall with temperature also being important in highland regions.^4^ Rainfall creates breeding sites for female mosquitoes to lay eggs, whereas humidity is key to adult mosquito daily survival. Temperature affects many aspects of malaria transmission dynamics, including both the development rates and survivorship of the juvenile and adult forms of the vector mosquitoes as well as the development rates of malaria parasites.^5^ Although *Plasmodium falciparum* is the dominant parasite across the continent, *Plasmodium vivax* is a significant cause of disease in sub-tropical and even temperate regions in Africa.^6^ This parasite is commonly the cause of malaria disease in the Ethiopian highlands,^7^ where it thrives in climates that are cooler than those suitable for *P. falciparum*.^8^ Season-to-season and year-to-year fluctuations in climate (climate variability) can have a significant impact on malaria transmission^9^ as can long-term trends in climate, which may or may not be associated with anthropogenic climate change.^10^ The impact of climate on entomological inoculation rates, prevalence, and morbidity/mortality are most strongly observed in regions of low and moderate seasonal transmission (outside of the humid zone).^11^

### Rationale for using climate information in malaria evaluations.

Central to any health impact assessment in an observational study is the concept of a baseline year or baseline period against which changes in outcomes can be measured. If the outcome is climate-sensitive we might expect that climate of the baseline year(s) chosen for evaluation purposes may have a substantial impact on the outcome of the evaluation. If malaria control intervention scale-up follows an unusually wet and warm baseline period and malaria incidence declines following interventions (during a drier and or cooler period), it may be tempting to attribute all of the decline in malaria outcomes to the investments in malaria control. However, correct attribution is important. As climate varies naturally over time, it is likely that the situation will at some point reverse (at least in part), resulting in an increase in climate suitability for transmission risk. If climate is not accounted for then higher malaria cases observed may be inappropriately attributed to program failure or other factors. Increasingly, climate data are being sought to remove this effect.^12–16^

Below are five observations, given as examples, which indicate that climate variability and trends potentially impact the attribution of reductions in malaria morbidity and mortality solely to interventions.

1. Declines in vector populations (*An. gambiae* s.str and *Anopheles funestus*) have occurred independently of any known specific malaria intervention—for example, in Tanzania.^17^
2. Replacement of *An. gambiae* s.str with the more zoophilic and drought tolerant *Anopheles arabiensis.*^18^ For example, *An. gambiae* s.str. adult females from indoor collections predominated in Kisumu, Kenya, from 1970 to 1998 (ca. 85%). Beginning in 1999, when significant regional droughts occurred in eastern Africa,^19^ *An. gambiae* s.str. decreased proportionately relative to *An. arabiensis*, then precipitously declined to rarity coincident with increased bed net ownership as national bed net distribution programs commenced in 2004 and 2006.^20^
3. Declines in malaria morbidity and mortality have preceded interventions. These declines have occurred on Pemba Island, Tanzania,^21^ and in a diverse range of ecological settings in Africa.^22^
4. Declines in malaria morbidity and mortality have been shown to be associated with drought in, for example, Sudan and Eritrea^23,24^ and across the Sahel.^25^
5. Variations in monthly rainfall in rural Tanzania may be strongly associated with all-cause mortality (largely associated with malaria) with younger age groups and the elderly population more at risk.^26^

### Climate data for use in analysis.

The relative importance of climate to malaria transmission varies in different regions of Africa with semi-arid regions and upland/highland regions being most susceptible to climate-related impacts. Access to high-quality climate data at appropriate spatial and temporal resolutions and with national coverage and local relevance is key to the use of climate data in malaria impact assessment.

There are four types of climate data of potential use; 1) ground-based observations from meteorological stations—either as a point measurement or as a gridded surface, 2) climate proxies derived from satellite data, 3) proxies derived from combining observations with the output of global or regional climate models—for example, reanalysis products, and 4) climate indices, which represent regional or large-scale atmospheric or oceanic phenomena. Table 1 summarizes common data sources, their characteristics, relevance, strengths, and limitations for use in malaria impact assessment. Many of these data sources may also be accessed through the IRI Data Library†; a powerful tool that serves over 400 public domain climate-related datasets.^27^ High-resolution environmental data (e.g., Landsat) and satellite-based global climate and environmental data may be accessed through Google Earth Engine (https://earthengine.google.com). However, access to climate data is not sufficient by itself. Understanding the characteristics and quality of the data is critical to the proper integration of climate and environmental information in malaria studies.

**Table 1:** Climate data (rainfall and temperature) for use in malaria impact assessment—advantages and limitations

	Climate information	Source	Advantages	Limitations
Ground observations				
Rainfall	Ground observations globally available	GTS*	Freely available. Actual measurements of rainfall. Information highly valuable for impact assessment at the location of the station and for testing other climate products	Represent a very small percentage of the station data available at the national level. May not be quality controlled and data may be missing. Rainfall highly localized but representativeness for a larger area can be improved if data aggregated over space and time. Large regions with very poor or no data.
	Climatology gridded (interpolated) ground observations	WorldClim†	Freely available. Pan African coverage. Information relevant for large-scale impact analysis where climatology is sufficient.	Quality entirely dependent on number, spatial distribution, and quality of ground observations, which varies a lot in space and time.
	Time series gridded (interpolated) ground observations	UEA-CRU‡	Freely available. Pan African coverage. Information relevant for large-scale impact analysis.	Quality entirely dependent on number, spatial distribution, and quality of ground observations, which varies a lot in space and time. Significant decline in data in recent decades. Best used for large-scale national and regional analysis.
		GPCC§		
	Ground point observations locally available—hourly to monthly	National archives and monitoring observations of the national meteorological agency.	Relatively high national coverage. The main source of meteorological data obtained using stations established and maintained using WMO criteria. Local knowledge of the data can help with quality control.	Data often restricted by national meteorological agencies—need data access agreement. Data may not be quality controlled and some may be missing. Ask for meta data associated with data files when daily data is aggregated to weekly or monthly data.
Air temperature (minimum and maximum)	Ground observations globally available	GTS	Freely available – information. highly valuable for impact assessment at the location of the station and for calibrating and verifying other climate products	Represent a very small percentage of the station data available at the national level. May not be quality controlled and data may be missing. Temperature varies with orography. Representativeness for a larger area can be improved if elevation and lat/long are incorporated into data aggregated over space and time. Large regions with very poor or no data
	Climatology gridded (interpolated) ground observations for period 1960–1999	WorldClim	Freely available. Pan African coverage. Information relevant for large scale impact analysis where climatology is sufficient.	Quality entirely dependent on number, spatial distribution, and quality of ground observations, which will vary in space and time.
	Time Series ridded (interpolated) ground observations	UEA-CRU	Freely available. Pan African coverage. Information relevant for large scale impact analysis.	Quality entirely dependent on number, spatial distribution, and quality of ground observations, which will vary in space and time. Significant decline in data in recent decades. Best used for large-scale subnational, national, and regional analysis.
	Ground point observations locally available from hourly to monthly	National archives and monitoring observations of the national meteorological agency.	Relatively high national coverage. The main source of meteorological data obtained using stations established and maintained using WMO criteria. Local knowledge of the data can help with quality control.	Data often restricted by national meteorological agencies—need data access agreement. Data may not be quality controlled and some may be missing. Ask for meta data associated with data files when daily data is aggregated to weekly or monthly data.
Reanalysis				
Rainfall	Created via a “frozen” data assimilation scheme and model(s), which ingest all available observations every 6–12 hours	ERA-40¶	Freely available, Pan African coverage with high temporal resolution (6–12 hours).	Large spatial scale. Quality of data varies by space and time due to varying data inputs over the years. Very poor representation of rainfall at any scale.
		ERA-Interim‖		
		NCEP-DOE**		
		MEERA, †† and many more		
Temperature (minimum and maximum)	Created via a “frozen” data assimilation scheme and model(s), which ingest all available observations every 6–12 hours	ERA-40	Freely available, Pan African coverage with high temporal resolution (6–12 hours). Good representation of temporal and spatial changes in air temperature, which can be improved by temporal aggregation and combining with ground station data.	Large spatial scale. Quality of data varies in space and time due to varying inputs.
		ERA-Interim		
		NCEP-DOE		
		MEERA, and many more		
Satellite climate data				
Rainfall	Weather monitoring satellites with global coverage	Satellite-based rainfall estimates (some combine satellite and limited station data from global archives/GTS)	Freely available. Pan African coverage. Provides a very good approximation of the spatial distribution of rainfall at the country or sub national level. Has high spatial and temporal resolution—for example, 4 km and daily.	Relationship to observed rainfall may vary according to orography and other local characteristics. May not capture rainfall extremes well. Quality dependent on calibration and integration of observed station data.
		RFE2,‡‡ ARC§§ TAMSAT¶¶	Representation of actual rainfall at local scale is best over areas where convective rainfall is dominant	
		CMAP‖‖ CHIRPS,*** etc.		
LSTs and estimated minimum air temperatures	LSTs derived from thermal sensors	MODIS LST††† during the night	Freely available, Pan African, high spatial resolution (1 km).	Relationship to observed temperature may vary according to land cover and other local characteristics. May not capture air temperature well. Quality dependent on calibration and integration of observed station data.
ENACTS‡‡‡	Blended product rainfall	Combines all quality controlled national station data with best globally available satellite product	High spatial and temporal resolution (4–5 km and daily) for over 30 years with much higher accuracy than other products as it incorporates data from the national observations archive and monitoring data. Suitable for analysis at national, sub-national, and local level. Derived climate products available on national meteorological agency websites	Quality varies according to the number and quality of observations used to calibrate and integrate into data set. ENACTS climate product data may be restricted by national meteorological agencies—need data access agreement.
	Blended product temperature	Combines all quality controlled national station data with best globally available elevation and reanalysis products	High spatial and temporal resolution (4–5 km and 10 daily) for over 30 years with much higher accuracy than other products as it contains the national observations archive and monitoring data. Suitable for analysis at national, sub-national, and local level. Climate products available on national meteorological agency website	Quality varies according to the number and quality of observations used to calibrate and integrate into data set. ENACTS climate product data may be restricted by national meteorological agencies—need data access agreement.

ARC = Africa Rainfall Climatology; CHIRPS = Climate Hazards Group InfraRed Precipitation with Station Data; CMAP = CPC Merged Analysis of Prediction; CRU = Climate Research Unit; DOE = Department of Energy; ENACTS = Enhanced National Climate Services products; GPCC = Global Precipitation Climatology Center; GTS = Global Telecommunications System; LST = Land Surface Temperature; MODIS = Moderate Resolution Imaging Spectroradiometer; NCEP = National Centers for Environmental Prediction; RFE = Rainfall Estimates; UEA= University East Anglia;

* Available at: www.wmo.int/pages/prog/www/TEM/GTS/index_en.html.

† Available at: www.worldclim.org.

‡ Available at: www.cru.uea.ac.uk/cru/data/hrg/.

§ Available at: climatedataguide.ucar.edu/climate-data/gpcc-global-precipitation-climatology-center.

¶ Available at: www.cgd.ucar.edu/cas/catalog/reanalysis/ecmwf/era40/sfc_mmeans.html.

‖ Available at: climatedataguide.ucar.edu/climate-data/era-interim.

** Available at: www.esrl.noaa.gov/psd/data/gridded/data.ncep.reanalysis2.gaussian.html.

†† Available at: http://iridl.ldeo.columbia.edu/expert/SOURCES/.NASA/.GSFC/.MERRA/.

‡‡ Available at: http://www.cpc.ncep.noaa.gov/products/fews/rfe.shtml.

§§ Available at: http://www.cpc.ncep.noaa.gov/products/fews/AFR_CLIM/afr_clim.shtml.

¶¶ Available at: http://www.met.reading.ac.uk/∼tamsat/data/rfe_anom.html.

‖‖ Available at: http://www.cpc.ncep.noaa.gov/products/global_precip/html/wpage.cmap.html.

*** Available at: http://chg.geog.ucsb.edu/data/chirps/.

††† Available at: http://modis-land.gsfc.nasa.gov/temp.html.

‡‡‡ Available at: http://iri.columbia.edu/resources/enacts/.

It should be noted that the quality of globally available blended satellite, reanalysis, and ground station products depends critically on the quality, number, and distribution of the ground-based observations used in their calibration and creation. Access to station data is limited by the policy, cost recovery issues, and technical constraints of the National Meteorological and Hydrological Services (NMHS).^28^ Unfortunately, few countries in Africa have quality-assured climate information readily available and accessible at an appropriate spatial and temporal scale and so there is, by necessity, considerable reliance on often poorly calibrated and unvalidated global products (e.g., from satellite data or reanalysis).

### Integrating climate into specific malaria analyses.

Climate and environmental information have been used in a number of studies to help assess the impact of malaria interventions. Analyses range from a simple assessment of the climate pre- and post-intervention^16,29^ to using climate predictors with malaria mortality,^30^ cases,^23^ or slide positivity^31^ as the predicted outcome. Incorporating both temperature and rainfall along with other environmental or socioeconomic variables into geospatial analyses using a Bayesian statistical modeling approach with malaria as the dependent variable is increasingly seen as the way forward for modeling spatiotemporal disease risk.^32–36^ The use of climate-driven mathematical malaria models is another approach.^37–39^ However, to-date, there is insufficient evidence that they can be used in malaria impact assessments at relevant spatial and temporal scales such as district level monthly analysis across multiple epidemiological zones (epidemic, endemic, etc.).

Herein, we present an approach that can be used when high-quality, long-time series malaria data is lacking but high-quality climate data such as rainfall and temperature are available.^40^ Using specific operational tools that can incorporate climate data the approach is based on the general assumption that a warmer, wetter climate will likely increase the suitability for malaria transmission risk, whereas a drier, cooler climate will reduce it. In the absence of interventions or other social changes, we expect this to have a significant impact on malaria prevalence in areas where certain months of the year are deemed unsuitable for malaria transmission because it is too dry or too cool. We then assess the impact of choice of baseline years against which to compare intervention impact.

## MATERIALS AND METHODS

### Analysis of climate suitability for malaria transmission during pre- and post-intervention periods.

To overcome the constraints identified for quality control and relevance of global climate products, the International Research Institute for Climate and Society (IRI), with support of the President's Malaria Initiative and other partners, has pioneered a new approach referred to as “Enhancing National Climate Services (ENACTS).”^28,41,42^ Through this initiative, the availability of climate data is improved by careful quality control of data from the national observation network and combining all relevant station observations with satellite estimates for rainfall, and digital elevation models and reanalysis products for temperature. The new, quality assessed, spatially, and temporally complete (> 30 years for rainfall and > 50 years for temperature of 10-day data at 4–5 km^2^) ENACTS data products created allow for characterization of climate risks at a local scale with national coverage.

**Figure 1.**
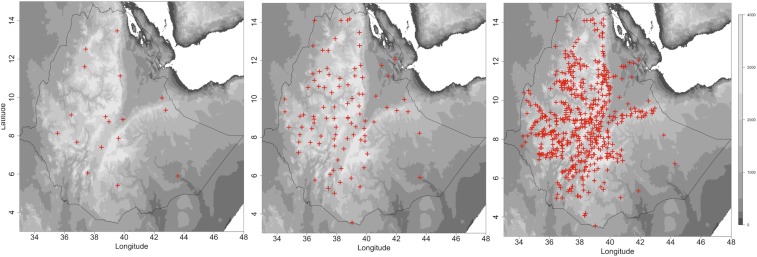
Observed rainfall gauge data made available by the Ethiopian National Meteorological Agency to (**A**) the Global Telecommunications System of the World Weather Watch, (**B**) Enhanced National Climate Services products (ENACTS) monitoring products, and (**C**) ENACTS historical products.

Developed rainfall and temperature (minimum and maximum) data and derived products are disseminated on the meteorological agency website^27,43^ and through other means (i.e., on request via e-mail). The ENACTS process provides a substantive improvement on the number and quality of ground observations incorporated into blended historical and monitoring rainfall estimates and temperature products currently available (Figure 1).

**Figure 2.**
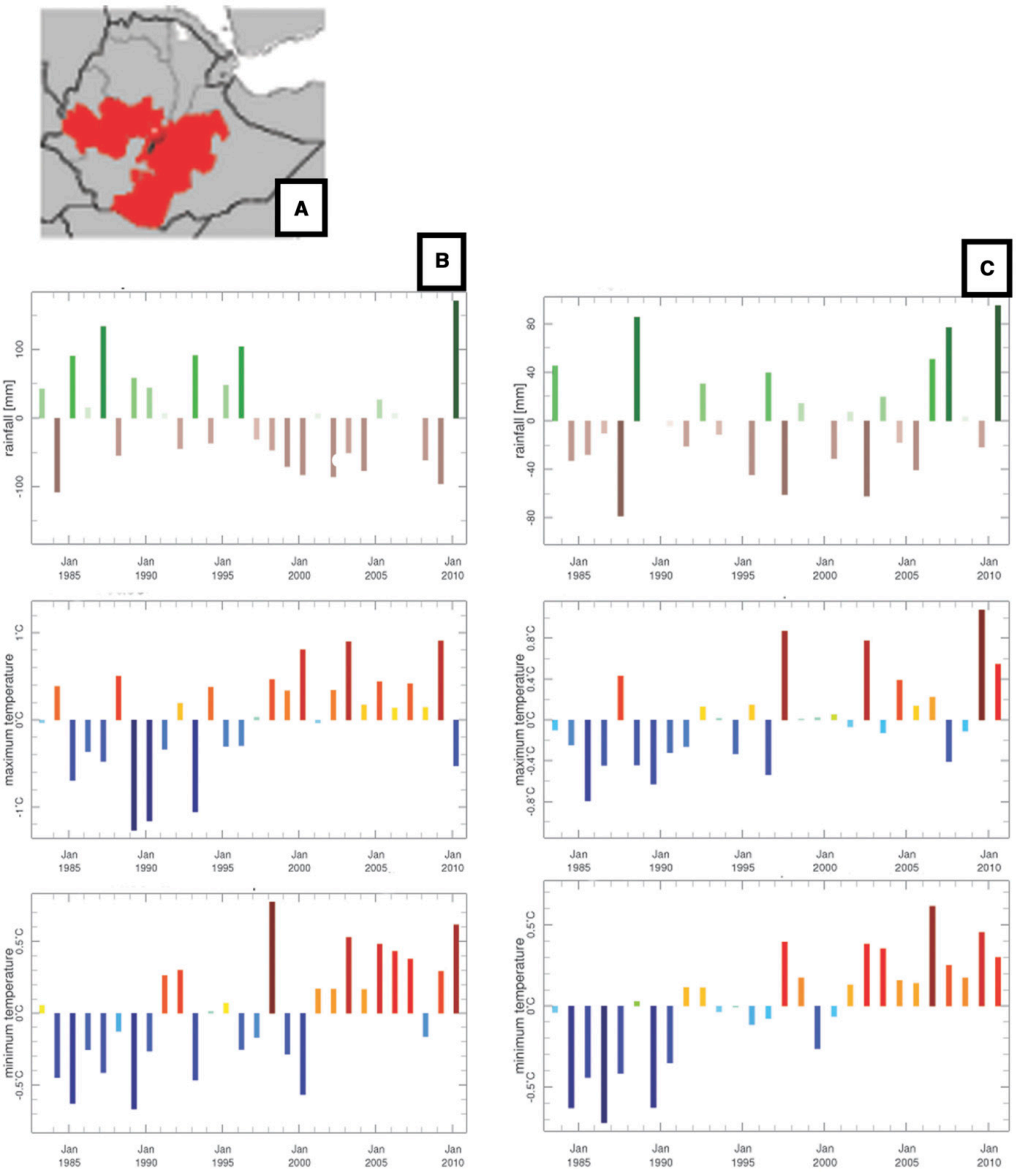
Outputs of National Meteorological Agency Enhanced National Climate Services products (ENACTS) Maproom Climate Analysis tool used to examine recent climate trends for the two rainy seasons-the Belg (left column) and Meher/Kiremt (right column) for (**A**) Oromia region Ethiopia; (**B**) trend in rainfall (top) max T (middle) and min T (bottom) for the Belg rainy season (February to May); (**C**) trend in rainfall (top) max T (middle) and min T (bottom) for the Kiremt rainy season (June to September). Similar information can be created using the ENACTS tool at region, zone, or woreda level.

In our study, monthly rainfall and temperature (minimum and maximum) data at 4–5 km resolution were obtained from the National Meteorological and Hydrological Services through the ENACTS initiative in Tanzania, Zanzibar, Rwanda, Ethiopia, and Mali.^28^ Representations of the data—for example, climatology, anomalies, trends, and derived products are available online in “Maprooms” on the meteorological agency websites. Figure 2A–C presents an example of long-term anomaly time series produced for rainfall and temperature by the Ethiopian ENACTS Climate Analysis tool. An extended drought period from 1997 to 2009 for the Belg season can be observed. The year 2010 was extremely wet in both seasons and also had high minimum temperatures (compared with the long-term average). Substantial warming trends in minimum and maximum temperature can be observed in both seasons over the period 1983–2010. Similar analysis are readily undertaken using Maprooms in all countries where ENACTS has been implemented (Ethiopia, Tanzania, Zanzibar, Madagascar, Rwanda, Ghana, Zambia, and Mali). In addition AGRHYMET—based in Niger (http://cradata.agrhymet.ne/maproom/) has developed a regional ENACTS rainfall product for the Sahel, which incorporates approximately 500 operational weather stations from the participating countries.

Malawi, Angola, Uganda, and Mozambique are all countries where ENACTS products and services are absent or in development. Senegal had regional ENACTS rainfall data and this was compared with global rainfall products. For these countries, where quality rainfall and temperature information for use at local level is difficult to access, national level climate analysis were undertaken using rainfall derived from the Merged Analysis of Precipitation from the Climate Prediction Center (CMAP); a global product that is freely available. Previous work has shown that in Botswana, CMAP provides a reasonable approximation of observed rainfall at the national and seasonal time scale.^9^ We compared the results with Climate Hazard Group Infrared Precipitation (CHIRPS) rainfall estimate that involves satellite data merged with archived station data that has recently been made available via the U. S. Geological Survey.^44^ This product is available for the entire globe at 5 km resolution but, as with other satellite-based data sources, the quality varies by country based on differing access to ground observations from the national meteorological and hydrological services. Both global products were tested against ENACTS data products, which are considered “gold standard” as they include all relevant climate data managed by the NMHS’s.

In addition, we explored options for accessing time series of minimum and maximum temperature that could be used to compare the Climate Suitability for Malaria Transmission (CSMT) pre- and post-intervention. Vancutsom and others^45^ explored the possibility of retrieving high-resolution minimum air temperature data from the Moderate Resolution Imaging Spectroradiometer (MODIS) over different ecosystems in Africa. Comparisons between night MODIS Land Surface Temperature (LST) data with minimum air temperature showed that MODIS night-time products provide a good estimation of the seasonal pattern of minimum air temperature over different ecosystems; however, year-to-year variability was not explored. They did show that air time maximum temperature LST was very poorly related to seasonal patterns in maximum air temperature. Because of these limitations and because MODIS LST are only available since 2000 and so are not suitable for most of the analysis considered in this manuscript (as preintervention periods often included time before 2000) they were not considered further. To assess the historical climatology of temperature in different African countries, we used the University of East Anglia monthly gridded average air temperature surfaces climatology (1951–2000).^46^ However, this data source was not suitable for the periods of our analysis; so instead, we accessed minimum and maximum temperature from reanalysis data obtained from National Aeronautics and Space Administration Goddard Space Flight Center MEERA‡ after exploratory analysis indicated it was a good proxy at large spatial scales. We were able to access and analyze the global products through the IRI Data Library.

Specific tools for use by the malaria control community that are currently delivered through the ENACTS websites (http://iri.columbia.edu/resources/enacts/) in Tanzania (including Zanzibar), Rwanda, Ethiopia, Rwanda, and Mali include

1. CSMT tool^46^ and
2. Climate Analysis tool.^28^
3. Weighted Anomaly Standardized Precipitation (WASP) tool.^47^

The CSMT tool was developed originally as a decision tool to support the timing of activities associated with malaria interventions in Africa.^48^ It is an interactive map that displays for each month the percent occurrence of years when climate conditions were suitable for malaria based on the ∼30-year historic ENACTS data. Climatic conditions are considered to be suitable for transmission when the monthly precipitation accumulation is at least 80 mm, the monthly mean temperature is between 18°C and 32°C (*P. falciparum*) and the monthly relative humidity is at least 60%.^49,50^ A modified tool, which uses a 16°C threshold has been developed to represent climate suitability for *P. vivax* malaria transmission. It is a useful tool for identifying where and when rainfall or temperature is likely a constraint to malaria transmission.

The Climate Analysis tool presents the history of rainfall and temperature data for any location or region as a time series of anomalies. This is particularly useful for temperature analysis where unusual increases in minimum temperature may increase the likelihood of malaria in highland regions identified as sensitive to temperature using the CSMT tool.

The original purpose of the WASP tool was to provide a simple visual means of relating country-averaged rainfall to a reference period of interest to explore the spatial and temporal patters of meteorological drought.^47^ To compute the WASP index, monthly precipitation departures from the long-term average are obtained and then standardized by dividing by the standard deviation of monthly precipitation. The standardized monthly anomalies are then weighted by multiplying by the fraction of the average annual precipitation for the given month. These weighted anomalies are then summed over a 12-month period in this case, and this result is itself standardized.

WASP index values for use in malaria analyses were originally developed using rainfall data from CMAP^47^ (https://iridl.ldeo.columbia.edu/maproom/Health/Regional/Africa/Malaria/MDG/index.html). We made a similar tool to analyze CHIRPS data at the national level and the tool was adapted for national/subnational use when high-quality ENACTS data became available. The latter can be found on the ENACTS Maproom sites at the participating NMHSs (iri.columbia.edu/resources/enacts).

For countries without ENACTS, we used CMAP and CHIRPS rainfall and MEERA reanalysis temperature accessed and analyzed through the IRI Data Library. In addition, we investigated climate trends and anomalies for Senegal using regional ENACTS data and global products. Using the CSMT tool from the IRI Data Library.^27^ We identified Malawi, Uganda, and Angola as countries where warming temperature may play a role in favoring malaria transmission were further explored using reanalysis temperature data (minimum and maximum).

### Comparison of different rainfall products for use at the national scale.

A comparable WASP analysis using the globally available CMAP and CHIRPS and ENACTS products rainfall products for Tanzania was undertaken (analysis not shown). Although showing clear similarities with the other products, CMAP rainfall (which includes limited station data from the Global Telecommunications System), did not capture well extreme rainfall including that of 1997/1998. Tanzania CHIRPS data appear very similar to the ENACTS national data when analyzed at the national level despite the fact that this product accesses fewer ground observations. Differences were observed at the subnational level (not shown). Based on these results, we used CHIRPS data whenever national ENACTS rainfall data were not available.

Of the 10 countries included in this study, six countries/regions (Tanzania, Zanzibar, Ethiopia, Rwanda, and Mali) had access to high-resolution ENACTS data for both rainfall and temperature. Regional ENACTS data were available for Senegal for rainfall alone and so both ENACTS and global products were used there. Analysis for an additional four countries (Malawi, Uganda, Mozambique, and Angola) was dependent on global rainfall and temperature products only.

## RESULTS

### Analysis of climate suitability for malaria transmission pre- and post-intervention.

**Figure 3.**
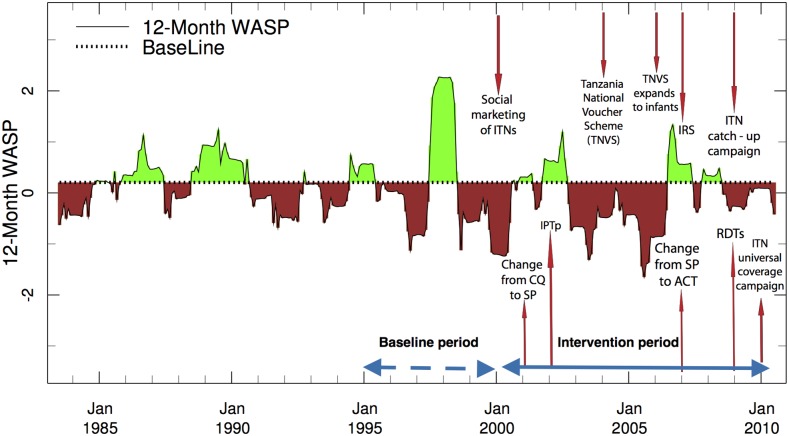
Tanzania ENACTS Weighted Anomaly Standardized Precipitation (WASP) Index using Enhanced National Climate Services products blended station and satellite data for Tanzania using a baseline period January 1995 to December 1999. Brown indicates time where rainfall was below the baseline average, whereas green indicates rainfall was above the baseline average. The WASP analysis is overlaid with the timing of interventions described in detail in Smithson and others (2015).

*Tanzania:* the ENACTS-based WASP rainfall analysis for Tanzania using a baseline period of 1995–1999 and associated interventions for the 2000–2010 is presented in Figure 3. The baseline period (1995–1999) used in the analysis by Smithson and others^15^ includes rainfall associated with the 1997/1998 El Niño and the early part of the intervention period 2000–2005 includes three major droughts, making it highly plausible that the impact of interventions will be overestimated. Only a few highland regions of Tanzania are likely to be impacted by the observed warming in minimum and maximum temperature that can be seen in the ENACTS temperature products.^29^

However, interventions in Tanzania were substantially increased post-2005, and therefore an evaluation comparing 1995–1999 and 2006–2010 may be more appropriate. If this new intervention period is chosen, then the intervention years are less likely to be influenced by the choice of an unusually wet (1995–1999) baseline period. Evidence of successful control against a more difficult climate increases the plausibility^2^ that the interventions are having a significant effect.

**Figure 4.**
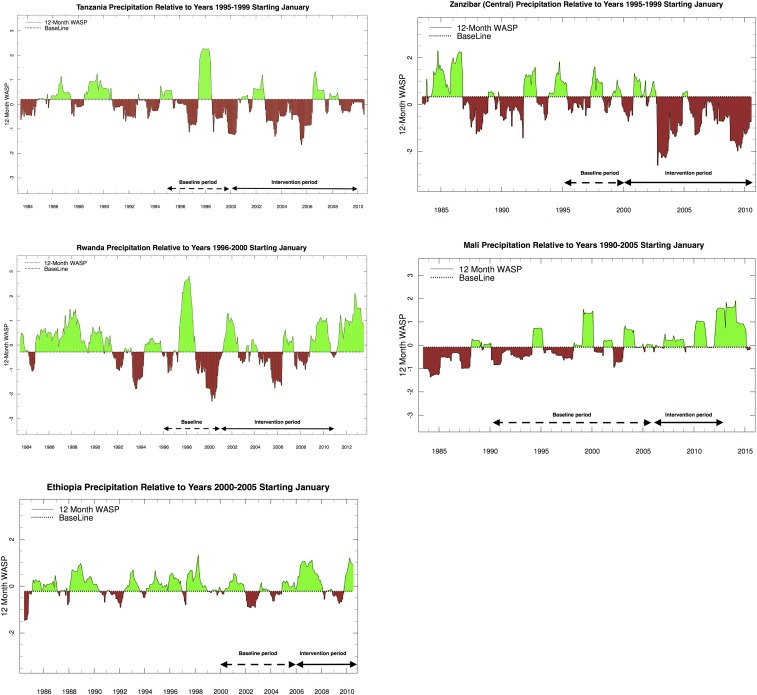
Enhanced National Climate Services products (ENACTS) Weighted Anomaly Standardized Precipitation rainfall analysis using varied baselines: (**A**) Tanzania 1995–1999 baseline, (**B**) Zanzibar Central 1995–1999 baseline, (**C**) Rwanda ENACTS 1996–2000 baseline, (**D**) Ethiopia ENACTS 2000–2005 baseline, and (**E**) Mali 1990–2005 baseline.

**Figure 5.**
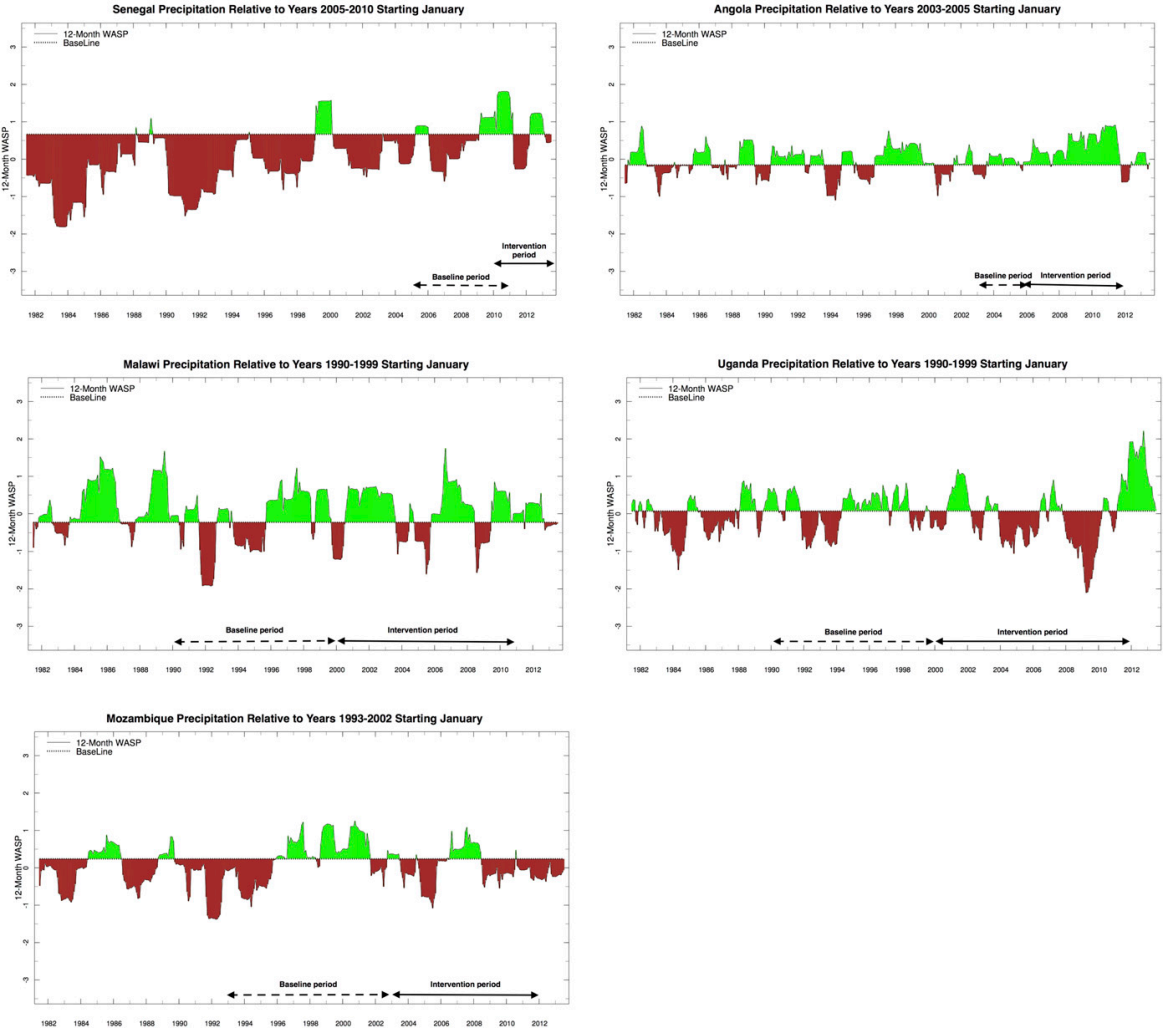
Climate Hazards Group InfraRed Precipitation with Station Data Weighted Anomaly Standardized Precipitation rainfall analysis using varied baselines: (**A**) Senegal 2005–2010 baseline, (**B**) Angola 2003–2005 baseline, (**C**) Malawi 1990–1999 baseline, (**D**) Uganda 1990–1999 baseline, and (**E**) Mozambique 1993–2002 baseline.

Similar analysis has been done for other countries and regions using both ENACTS and CHIRPS data as available (Figures 4A–E and 5A–E).

#### Zanzibar.

Although it has a generally wetter climate than mainland Tanzania (Figure 4A), rainfall variability in Zanzibar Island over the three decades (1982–2014) shows similar characteristics. Between 2000 and 2010, the island had a significant decline in rainfall with some extreme drought years.^51^ These droughts may have significantly assisted the early decline in malaria observed on the island,^52^ including the central region (Figure 4B).

#### Rwanda.

A similar analysis for Rwanda indicated that the evaluation baseline period of 1996–2000 was also dominated by the impact of the 1997/1998 El Niño (Figure 4C) and that there is also a risk that the evaluation will overestimate the impact of control measures during the intervention period. However, the baseline period also includes an extreme drought in 1998/1999, which exceeds any observed in intervention years. Changing the baseline period to 2000–2005 and the intervention period to 2006–2010 would suggest that climate-related risks have increased during the later period.

#### Ethiopia.

A recent study by Aregawi and others observed that malaria cases and deaths in Ethiopian hospitals decreased substantially during 2006–2011 relative to a baseline period 2000–2005 in conjunction with scale-up of malaria interventions.^16^ The authors observed that the decrease could not be accounted for by changes in hospital visits or malaria diagnostic testing, and an analysis of ENACTS climate data using WASP and other analysis tools was undertaken. It must be noted, however, that given the complexity of the Ethiopian climate a national analysis may obscure many important sub-national variations.^12^ Using the Ethiopia ENACTS WASP products extracted for 14 homogeneous rainfall zones of Ethiopia,^53^ climate analysis over the period—July 1984 to June 2010—was undertaken to assess if pre- or postintervention periods were likely to be influenced by drought. The results from the ENACTS WASP indicate that for most regions there was lower rainfall in the pre-intervention (baseline) period (2000–2005) relative to the postintervention period (2006–2010), Figure 4D.^16^ In addition, analysis of temperature using the Climate Analysis tool (Figure 2A–C) indicates that the climate of Ethiopia has been generally getting warmer over the last 30 years particularly in highland areas (with considerable year-to-year variations). Approximately 50% of Ethiopia’s population live in highland areas that are deemed sensitive to climate-related changes in the risk of malaria. Higher temperatures and rainfall in the intervention period suggest that the climate suitability for malaria from 2006–2010 was above that of the baseline period. Thus, any large-scale declines in malaria cases and deaths during 2006–2010 are against a backdrop of increasing CSMT.

#### Mali.

The Sahel suffered extreme droughts in the 1970s and 1980s; and so the WASP tool for rainfall indicates that the CSMT has likely improved the last two decades (Figure 4E). As a consequence, the intervention period (2006–2011) is considerably wetter than the pre-intervention period (1990–2005) suggesting that there is a risk that the impact of interventions will be underestimated. Temperature data also indicate general warming over the period, although there is no evidence that temperature is currently a constraint to transmission in the Sahel.

Five countries were without ENACTS products and services and we used global rainfall data instead. Using the climate-based CSMT tool from the IRI Data Library, we indentified Malawi, Uganda, and Angola as countries where warming temperature may play a role in increasing climate suitability for malaria risk, and as a result trends in temperature in these countries were explored using MEERA reanalysis data.

#### Senegal.

The WASP tool using ENACTS was not available for this analysis, so a simple anomaly for the main rainy season was used instead. This was then compared with a CHIRPS-based WASP and the results were found to be highly correlated. The results from both analyses indicate strong positive trends in rainfall over the last 30 years (Figure 5A), indicating that the CSMT has likely increased in recent years, suggesting that there is a risk that the impact of interventions will be underestimated. Temperature data is not available through ENACTS at AGHRYMET; however, MERRA reanalysis data suggest that, in line with the rest of Africa, Senegal has been warming over the last 30 years. This warming is unlikely to have increased malaria suitability across this region as temperatures are already optimum.

#### Angola.

CHIRPS WASP analysis indicates climate suitability for malaria is higher for the intervention period (2006–2011) relative to the baseline period (2003–2005) (Figure 5B). As Angola has recently emerged from civil war, the quality of the climate data available for integration into global products is low.

#### Malawi.

CHIRPS WASP analysis indicates little difference in rainfall between the 2000–2010 intervention period and the 1990–1999 baseline (Figure 5C), although warming temperatures in the highland regions may have increased CSMT. However, global products for Malawi do not include substantial ground observations and the quality is likely poor. The lack of observed decline in malaria cases and mortality^54^ are unlikely to be associated with a significant increase in climate suitability for malaria during the intervention period.

#### Uganda.

CHIRPS WASP analysis indicates that the intervention period 2000–2011 was dry relative to 1990–1999 and included three significant drought periods but no indication that the 1997/1998 El Niño had a significant impact on rainfall (Figure 5D). Even with the observed warming trend in temperature, the CSMT in the intervention period is likely less than that of the baseline.

#### Mozambique.

There was little observed change in rainfall between baseline period (1993–2002) and intervention period (2003–2011) (Figure 5E).

**Figure 6.**
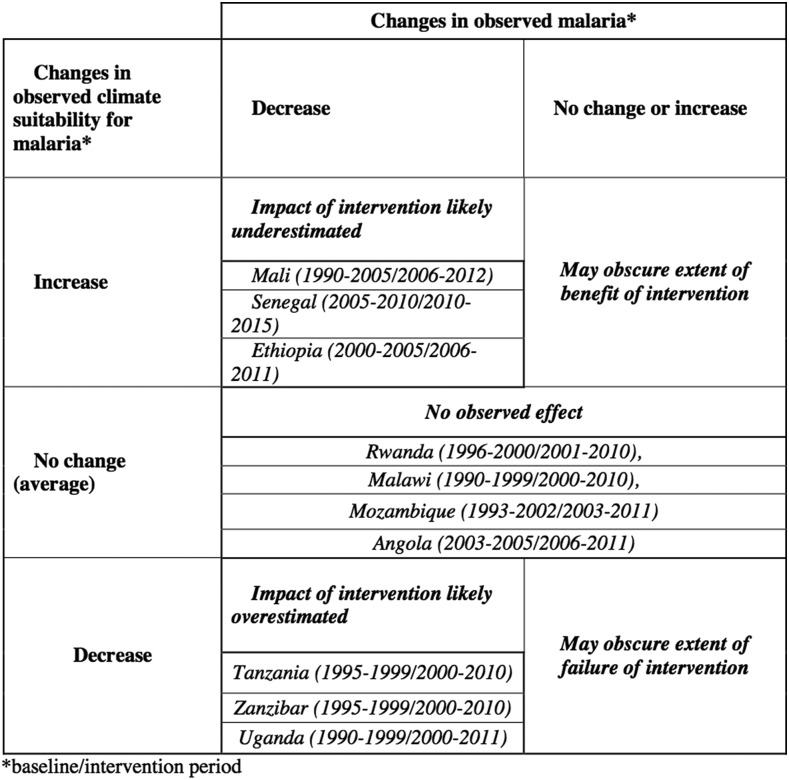
Possible outcomes if climate is not factored into malaria impact assessment—including country results.

In summary (Figure 6, Table 2), we found that three countries/regions (Tanzania, Zanzibar, and Uganda) likely had a more suitable climate during the baseline year rather than post-intervention, suggesting a significant risk in overestimating the impact of interventions in these eastern African countries. For Tanzania, Zanzibar, and Rwanda, the pre-intervention periods were strongly impacted by the 1998/1998 El Niño, which was associated with extreme rainfall and malaria outbreaks. Meanwhile, Ethiopia (a country that straddles the climate of eastern Africa and the Sahel), Mali and Senegal all had climates that were more favorable to malaria transmission after the intervention period based on rainfall alone. As malaria transmission in Senegal and Mali in populated areas is not constrained by temperature, no further consideration was made of this climate driver. However, Ethiopia and Malawi have upland and highland regions and variability in temperature is an important consideration.

**Table 2:** Data, methods, and results of climate analysis for each country/region

Country	Spatial scale	Climate data used	Malaria constrained by temperature (assessed using CSMT)	Baseline period	Intervention period	Type of analysis	Results*
Tanzania	District, region, country	ENACTS rainfall and ENACTS temperature	Yes	1995–1999	2000–2010	WASP, temperature anomaly analysis	Climate likely to have influenced the decline in malaria in the first half of the intervention period (2000–2005) relative to the baseline but not the second half—2006–2010 relative to the first. Base line period includes 1997/1998 El Niño
Zanzibar	District, Island	ENACTS rainfall and ENACTS temperature	No	1995–1999	2000–2010	WASP	Climate likely to have influenced the decline in malaria in the first half of the intervention period (2000–2005) relative to the baseline but not the second—2006–2010. Base line period includes 1997/19988 El Niño
Rwanda	District, region, country	ENACTS rainfall and ENACTS temperature	Yes	1996–2000	2001–2010	WASP, temperature anomaly analysis	The Rwanda country WASP indicates that the malaria baseline period of 1996–2000 was dominated by the impact of the 1997/1998 El Nino. However, drought in 1999 suggests that no major difference between baseline and intervention period.
Ethiopia	Climate regions, District, region, country	ENACTS rainfall and ENACTS temperature	Yes	2000–2005	2006–2011	WASP, temperature anomaly analysis	Climate suitability for malaria is higher after intervention period relative to baseline. El Nino impact varies across country.
Senegal	Country	ENACTS and CHIRPS rainfall and REANALYSIS temperature	No	2005–2010	2010–2015	Time series rainfall and temperature anomaly analysis	Climate suitability for malaria is higher during intervention period relative to baseline. Long-term wetting trend.
Mali	District, region, country	ENACTS rainfall and ENACTS temperature	No	1990–2005	2006–2012	WASP and temperature anomaly analysis	Climate suitability for malaria is higher during intervention period relative to baseline. Long-term wetting trend.
Angola	Country	CHIRPS rainfall and REANALYSIS temperature	Yes	2003–2005	2006–2011	WASP and temperature anomaly analysis	No clear change in rainfall between baseline and intervention period. Clear warming during period of intervention.
Malawi	Country	CHIRPS rainfall (WASP) and REANALYSIS temperature	Yes	1990–1999	2000–2010	WASP and temperature anomaly analysis	Climate suitability for malaria is similar during intervention period and baseline.
Uganda	Country	CHIRPS rainfall and REANALYSIS temperature	Yes	1990–1999	2000–2011	WASP and temperature anomaly analysis	Climate suitability for malaria is lower during intervention period than during baseline.
Mozambique	Country	CHIRPS rainfall and REANALYSIS temperature	No	1993–2002	2003–2011	WASP and temperature anomaly analysis	Climate suitability for malaria is similar during intervention period and baseline.

CSMT = Climate Suitability for Malaria Transmission; ENACTS = Enhanced National Climate Services products; WASP = Weighted Anomaly Standardized Precipitation; CHIRPS = Climate Hazards Group InfraRed Precipitation with Station Data.

* Countries showed general warming trend over the last 30 years using REANALYSIS temperature data.

Data collected from Rwanda, Mozambique, Malawi, and Angola did not indicate a substantial shift in rainfall between baseline and intervention periods. As elsewhere, reanalysis data indicated long-term warming, but this is likely insufficient to substantially increase climate suitability for transmission pre- and post-intervention.

## DISCUSSION

**Figure 7.**
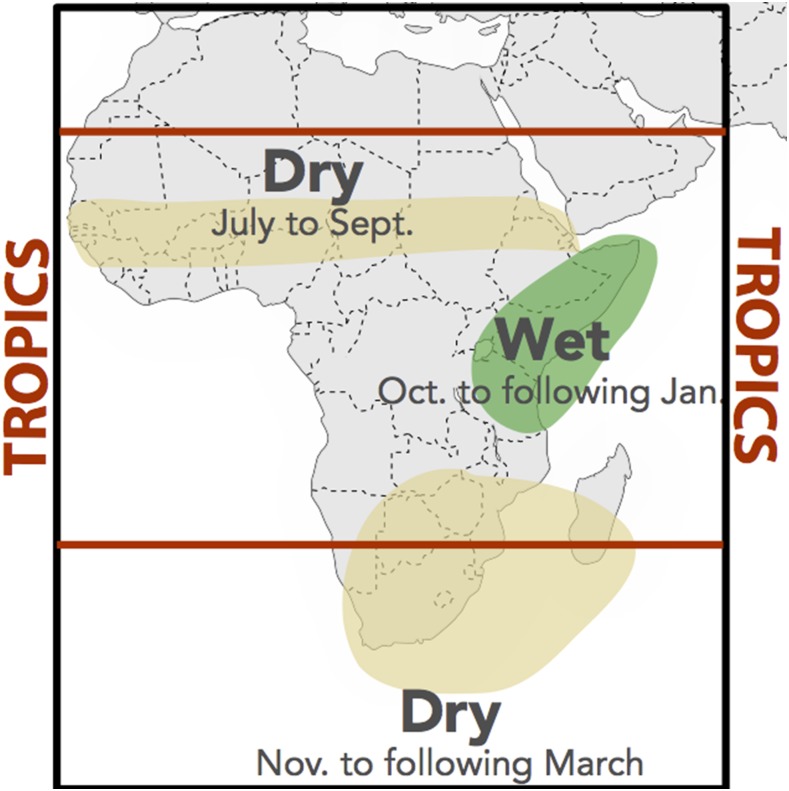
Likely impact of El Nino rainfall in Africa. In addition, general atmospheric warming occurs across the tropics during an El Nino event. Local temperature will be influenced by rainfall (https://iridl.ldeo.columbia.edu/maproom/IFRC/FIC/ElNinoandRainfall.pdf).

Climate varies at multiple spatial and temporal scales. The regional impact of ENSO in Africa illustrates some of the large-scale issues. For example, El Niño has a drying effect in the Sahel and southern Africa during their single rainy season but is associated with unusually heavy rains in eastern equatorial Africa (Figure 7) during the short rains.

### 

#### Eastern Africa.

This region has been plagued with an increased frequency of drought since 1999 (predominantly resulting from loss of rainfall in the main March to May rainy season associated with a shift in sea surface temperatures in the tropical pacific. ^55^) In our analysis, we observe the impact of the east African droughts (2000, 2003–2005, 2009) in the rainfall data for Tanzania, Zanzibar, Ethiopia, and Uganda.

Eastern equatorial Africa including Tanzania, Zanzibar, Ethiopia, and Rwanda is also impacted by the effects of El Niño, which results in higher than average rainfall in the October to December rainy season. Ethiopia straddles the climate patterns of eastern Africa and the Sahel, making national scale analysis challenging. By examining the different rainy seasons separately differences in the February to May season (Belg), which follows the eastern Africa March to April to May season and the July to September season (Kiremt) can be seen (Figure 2A–C). The third season—October to December is normally a low rainfall season; however, this can change during an El Niño year. In Ethiopia, both temperature and rainfall are very important drivers of malaria transmission; and overall, there has been a strong positive trend in climate suitability for transmission over the last 30 years—and more specifically during the intervention period relative to the baseline.

#### The Sahel

The climate of Senegal and Mali also reflects larger regional patterns. The Sahelian region is strongly impacted by decadal variability in rainfall with the last major drought cycle occurring in the 1970s and 1980s,^56^ which was associated with a large-scale decline in malaria.^25^ Since that time, the region has been getting wetter, and there is evidence that this shift in climate regimen has made a more favorable climate for malaria vectors in the region.^57^

#### Southern Africa.

The region’s climate is influenced by atmospheric circulations in both the tropics and the mid-latitudes. The main rainy season typically extends from October to April across much of the region, peaking during the southern-most extension of the inter-tropical convergence zone. Malawi and Mozambique straddle the climate regimes of eastern and southern Africa (Figure 7) and in these circumstances, a national analysis may miss strong trends in the north and south of the countries, which have different climate drivers. Assessing climatic trends in Angola is made difficult by the lack of national data resulting from many years of civil unrest and war. Global products therefore are likely poorly calibrated.

Challenges with data quality and quantity are significant constraints to the analysis undertaken here and elsewhere. A multi-country analysis of malaria from nationally representative surveys in Angola, Liberia, Mozambique, Senegal, and Rwanda sought to account for climate and environmental variability using global climate and environmental products rainfall, Normalized Difference Vegetation Index, altitude, LST night, LST day—acquired for the 6-month period prior to the survey). According to the authors, changes in climatic and environmental variables could not account for declines in malaria between first and second surveys.^58^ A recent study^59^ exploring the relative contribution of climate variability and vector control coverage to changes in malaria parasite prevalence in Zambia 2006–2012 has concluded that subnational changes in malaria parasite prevalence are highly influenced by climate over short time scales and must be accounted for in assessing the effectiveness of malaria control programs. The quality of the climate data in these studies and their correspondence to local observations was not assessed. In general, the effect of using an imprecise estimate of exposure to a climatic phenomenon for prediction purposes is to attenuate the corresponding regression parameter toward zero.^60^ Conversely, the better the data the more likely significant relationships will be observed.

The quality of climate data with national coverage and local relevance has improved in recent years with the arrival of ENACTS products (rainfall and temperature) and services at the national level and CHIRPS (rainfall) data at the global level. Access to locally relevant data with national coverage is important for many countries as malaria and climate are rarely homogenous across the country. Indeed in some countries, straddling different climate regimes (such as Malawi and Mozambique), a national analysis may obscure significant subnational variability and trends in climate drivers that act in opposite directions (e.g., northern Mozambique is likely wetter than average during an El Niño, whereas southern Mozambique is drier). In countries where ENACTS temperature data are available, warming of minimum and maximum temperatures at the national level may be observed but variations also apply at the subnational level. Reanalysis data indicate that there is consistent warming across the continent over the last 30 years.

Evaluations of the impact of interventions on malaria outcomes are undertaken using a plausibility argument.^1^ This is because the causal pathways between intervention and outcome are hard to identify and monitor and may be significantly influenced by other factors such as climate. In particular, climate may act as a significant factor in malaria impact evaluations in regions where seasonal rises in case numbers, epidemics, and long-term trends are climate sensitive. As a consequence, choice of baseline periods should be carefully assessed and variations in CSMT must be taken into account when effectiveness evaluation claims are made.

Herein, we have established an approach to assess the plausibility of climate acting to overestimate or underestimate the impact of malaria control interventions. To do this rigorously and routinely, NMCPs must have access to quality climate data with local relevance and national coverage. Furthermore, users must have robust methodologies and access to useful tools and ensure that they are available at the national level.^61^

Despite all the challenges with the data, in the absence of interventions, malaria is remarkably consistently related to climate in large regions of eastern, western, and southern Africa through its seasonal patterns and year-to-year variability. Removing the effect of climate by realigning baseline periods, where possible, provides a means to establish a more robust estimate of program effectiveness and can reassure donors and government that international and local resources are being used to maximum effect. However, to use climate data effectively, new collaborative engagements must be formed between the malaria and climate community. Such collaborations may extend opportunities for the use of climate in malaria programs to other climate-sensitive diseases.

## Footnotes

† Available at: http://iridl.ldeo.columbia.edu.

‡ Available at: http://iridl.ldeo.columbia.edu/expert/SOURCES/.NASA/.GSFC/.MERRA/.
